# Risk Factors for Hospitalization and Mortality due to COVID-19 in Espírito Santo State, Brazil

**DOI:** 10.4269/ajtmh.20-0483

**Published:** 2020-07-16

**Authors:** Rita de Cássia Menezes Soares, Larissa Rodrigues Mattos, Letícia Martins Raposo

**Affiliations:** 1 Department of Quantitative Methods, Center for Exact Sciences and Technology, Federal University of the State of Rio de Janeiro, Rio de Janeiro, Brazil;; 2 Department of Public Health Nursing, Anna Nery Nursing School, Federal University of Rio de Janeiro, Rio de Janeiro, Brazil

## Abstract

Brazil is, at the time of writing, the global epicenter of COVID-19, but information on risk factors for hospitalization and mortality in the country is still limited. Demographic and clinical data of COVID-19 patients until June 11th, 2020 were retrieved from the State Health Secretariat of Espírito Santo, Brazil. Potential risk factors for COVID-19 hospitalization and death were analyzed by univariate and multivariable logistic regression models. A total of 10,713 COVID-19 patients were included in this study; 81.0% were younger than 60 years, 55.2% were female, 89.2% were not hospitalized, 32.9% had at least one comorbidity, and 7.7% died. The most common symptoms on admission were cough (67.7%) and fever (62.6%); 7.1% of the patients were asymptomatic. Cardiovascular diseases (23.7%) and diabetes (10.3%) were the two most common chronic diseases. Multivariate logistic regression analysis identified an association of all explanatory variables, except for cough and diarrhea, with hospitalization. Older age (odds ratio [OR] = 3.95, *P* < 0.001) and shortness of breath (OR = 3.55, *P* < 0.001) were associated with increase of odds to COVID-19 death in hospitalized patients. Our study provided evidence that older age, male gender, Asian, indigenous or unknown race, comorbidities (smoking, kidney disease, obesity, pulmonary disease, diabetes, and cardiovascular disease), as well as fever and shortness of breath increased the risk of hospitalization. For death outcome in hospitalized patients, only older age and shortness of breath increased the risk.

## INTRODUCTION

Since December 2019, when groups of patients with viral pneumonia were confirmed to be infected with a novel coronavirus, named as SARS-CoV-2, COVID-19 has become a public health emergency of international interest.^1^

So far, on June 30, 2020, there have been 10,185,374 confirmed cases and 503,862 deaths worldwide.^2^ In Brazil, the first recorded case occurred on February 26, 2020, after a 61-year-old man from São Paulo, who returned from Italy, tested positive for SARS-CoV-2.^3^ To date, 1,404,041 cases have been confirmed and 59,594 deaths were registered in the country.^4^

Most COVID-19 patients present mild to moderate symptoms, but 14% of the cases become severe and 5% become critical.^5^ Fatality rates of 1.4^6^–4.3%^7^ have been reported in different regions or hospitals. So far, in Brazil, this rate is 4.3%.^4^ Clinical symptoms such as fever, cough, and shortness of breath have been described extensively in published literature. Comparing characteristics of patients who have died so far with those of the ones who have recovered, studies have found that the former were older, more likely to be male, and to have a comorbidity, such as hypertension, diabetes, cardiovascular disease, or respiratory disease.^5–9^ Other risk factors include obesity and smoking.^6,8^ Most of these studies are focused on Chinese data because China was the initial epicenter of the disease,^1,5^ which makes it necessary to assess risk factors in different population groups or settings.

Brazil is, at the time of writing, the global epicenter of the disease, and the causes for it are varied, but some influence can be attributed to late or inefficient lockdown/quarantines and inadequate governmental measures and guidance, giving the populace a false sense of security.^10^ As many third world countries, Brazil suffers from lack of access to resources such as beds, tests, and ventilators,^11^ and this scarcity, among many impacts, also facilitates disease spread and complications in patients.

Here, we present patient information acquired from the Health Secretariat of Espírito Santo state (Brazil) on June 11, 2020. Patients selected were confirmed for COVID-19 and had a definite clinical outcome (death or recovery). The present study analyses the relationships of several clinical factors, comorbidities, and demographic characteristics with hospitalization and death by COVID-19.

## METHODS

### Data collection.

This study included a cohort of SARS-CoV-2–infected patients from Espírito Santo state, Brazil. This state was chosen because it presented one of the best open data quality indexes for COVID-19^12,13^ and had clinical data for comorbidities and symptoms readily available. Data were downloaded from the State Health Secretariat page about COVID-19^14^ on June 11, 2020, last updated on June 11.

All patients who were diagnosed with COVID-19 between February 29, 2020 (i.e., when the first patients were admitted) and June 11, 2020, were included in our study, therefore, excluding cases considered to be still open. Patients were confirmed for COVID-19 if they had a positive laboratory examination result (real-time PCR or serological test) or if they were a suspected case that previously came into close contact or resided with someone with a confirmed laboratory diagnosis.^15^ A total of 24,428 COVID-19–confirmed cases were collected, and, of those, 15,109 had closed case status. Only patients with complete data for predictive variables were included in the study to avoid confounding by missing data, totaling 10,713 patients.

### Outcomes and predictors.

Epidemiological characteristics, clinical signs and symptoms, comorbidities, inpatient hospitalization, and survival status (recovery or SARS death) were obtained from this dataset.

Two separate outcomes were analyzed: hospitalization and death from COVID-19. For both of them, predictive factors were gender, age, race, comorbidities, and signs and symptoms.

Patients included in the study had been confirmed for COVID-19, recovered or died from this disease, had their case closed and had complete information for explanatory variables (gender, age, race, comorbidities, and signs and symptoms). For hospitalization, all those patients were analyzed and for death outcome, only those who had been hospitalized.

### Statistical analysis.

Descriptive statistics included frequency analysis (%) for all categorical variables. Chi-square test was used to compare differences between groups where appropriate. To explore the association of epidemiological and clinical characteristics and the risk for hospitalization or death in 2019-nCoV–infected patients, univariate and multivariate binary logistic regression models were performed.

We did not use variables on univariate analysis if their between-group differences were not significant. Variables with *P*-value < 0.05 in previous analysis were entered into logistic multivariate regression models. The backward step-down selection process with the Akaike information criterion was used to select variables to include in the final model. The odds ratio (OR) and the 95% CI were reported. *P*-value < 0.05 was considered statistically significant.

All statistical analyses were performed on R software (version 3.6.3, R Foundation for Statistical Computing, Vienna, Austria).^16^

## RESULTS

### General results and hospitalization outcome.

A total of 24,428 patients were confirmed with COVID-19 in the Espírito Santo state until June 11, 2020. Only patients who recovered or died, whose case was closed and with complete information for explanatory variables previously selected from the database, were included in this study, totaling 10,713 patients, with 821 deaths (7.7%); 81.0% of patients were younger than 60 years, 55.2% were female, and 89.2% were not hospitalized. Of the 10,713 COVID-19 patients, 3,523 (32.9%) had at least one comorbidity.

The signs or symptoms on admission for the 10,713 patients were cough (67.7%), fever (62.6%), headache (51.3%), runny nose (38.8%), sore throat (29.7%), shortness of breath (28.7%), and diarrhea (14.7%). Cardiovascular diseases (23.7%) and diabetes (10.3%) were the two most common chronic diseases among all 10,713 included patients.

Older patients had a higher percentage of males than younger ones (50.7% versus 43.5%), more Asian/indigenous/unknown race (27.0% versus 21.9% of younger patients), and less black/multiracial (38.2% versus 43.0% of younger patients). These associations were significant by chi-square analysis (*P* < 0.05). Also, older patients presented a higher prevalence of all comorbidities, except obesity (*P* = 0.068). They had a higher prevalence than expected of cough and shortness of breath, and less diarrhea, headache, runny nose, and sore throat (*P* < 0.05) than younger patients (data not shown). There was no association between age and presence of signs and symptoms (being asymptomatic) (*P* = 0.513).

As shown in Table 1, older (≥ 60 years, *P* < 0.001) and male (*P* < 0.001) patients were significantly more likely to be hospitalized from COVID-19 than others. Hospitalization status was also different by race (*P* < 0.001). All comorbidities and symptoms were individually associated with hospitalization (*P* < 0.05) (Table 1). Patients with the following symptoms were less hospitalized than expected: diarrhea, headache, runny nose, and sore throat. Other comorbidities and symptoms resulted in hospitalization rates greater than expected.

**Table 1:** Clinical characteristics of COVID-19 patients by hospitalization status

	Nonhospitalized	Hospitalized	*P*-value*
Total, *N* (%)	9,561 (89.2)	1,152 (10.8)	
Age (years), *n* (%)			< 0.001
< 60	8,130 (93.7)	546 (6.3)	
≥ 60	1,431 (70.3)	606 (29.7)	
Gender, *n* (%)			< 0.001
Female	5,415 (91.6)	494 (8.4)	
Male	4,146 (86.3)	658 (13.7)	
Race, *n* (%)			< 0.001
White	3,403 (90.8)	345 (9.2)	
Black/multiracial	4,060 (90.0)	453 (10.0)	
Asian/indigenous/unknown	2,098 (85.6)	354 (14.4)	
Comorbidities, *n* (%)			
Cardiovascular diseases			< 0.001
No	7,546 (92.3)	626 (7.7)	
Yes	2,015 (79.3)	526 (20.7)	
Diabetes			< 0.001
No	8,738 (90.9)	875 (9.1)	
Yes	823 (74.8)	277 (25.2)	
Kidney diseases			< 0.001
No	9,492 (89.7)	1,093 (10.3)	
Yes	69 (53.9)	59 (46.1)	
Obesity			< 0.001
No	9,077 (89.7)	1,039 (10.3)	
Yes	484 (81.1)	113 (18.9)	
Pulmonary diseases			< 0.001
No	9,151 (89.8)	1,041 (10.2)	
Yes	410 (78.7)	111 (21.3)	
Smoking			< 0.001
No	9,429 (89.8)	1,075 (10.2)	
Yes	132 (63.2)	77 (36.8)	
Signs and symptoms, *n* (%)			
Cough			0.013
No	3,122 (90.3)	334 (9.7)	
Yes	6,439 (88.7)	818 (11.3)	
Diarrhea			< 0.001
No	8,111 (88.8)	1,026 (11.2)	
Yes	1,450 (92.0)	126 (8.0)	
Fever			< 0.001
No	3,672 (91.7)	333 (8.3)	
Yes	5,889 (87.8)	819 (12.2)	
Headache			< 0.001
No	4,366 (83.6)	854 (16.4)	
Yes	5,195 (94.6)	298 (5.4)	
Runny nose			< 0.001
No	5,633 (86.0)	919 (14.0)	
Yes	3,928 (94.4)	233 (5.6)	
Shortness of breath			< 0.001
No	7,140 (93.5)	495 (6.5)	
Yes	2,421 (78.7)	657 (21.3)	
Sore throat			< 0.001
No	6,542 (86.9)	984 (13.1)	
Yes	3,019 (94.7)	168 (5.3)	

* *P*-values were calculated by chi-square test. *P*-value < 0.05 was considered statistically significant.

In the univariate analysis for hospitalization, the odds of COVID-19 hospitalization were higher in patients aged 60 years or older, male, and of Asian, indigenous, or unknown race (Table 2). All comorbidities increased the likelihood of hospitalization (OR > 1), as did cough, fever, and shortness of breath (Table 2). Diarrhea, headache, runny nose, and sore throat decreased the likelihood of hospitalization (OR < 1) (Table 2).

**Table 2:** Risk factors associated with hospitalization due to COVID-19

	Univariable OR (95% CI, *P*-value)*	Multivariable OR (95% CI, *P*-value)*
Age (years) (vs. < 60)		
≥ 60	6.31 (5.55–7.17, < 0.001)	3.40 (2.91–3.96, < 0.001)
Male gender (vs. female)	1.74 (1.54–1.97, < 0.001)	1.43 (1.25–1.65, < 0.001)
Race (vs. white)		
Black/multiracial	1.10 (0.95–1.28, = 0.202)	1.07 (0.90–1.26, = 0.443)
Asian/indigenous/unknown	1.66 (1.42–1.95, < 0.001)	1.47 (1.23–1.75, < 0.001)
Comorbidities		
Cardiovascular diseases	3.15 (2.77–3.57, < 0.001)	1.30 (1.11–1.53, = 0.001)
Diabetes	3.36 (2.88–3.91, < 0.001)	1.34 (1.10–1.61, = 0.003)
Kidney diseases	7.43 (5.20–10.56, < 0.001)	2.41 (1.59–3.66, < 0.001)
Obesity	2.04 (1.64–2.52, < 0.001)	1.74 (1.35–2.23, < 0.001)
Pulmonary diseases	2.38 (1.90–2.95, < 0.001)	1.46 (1.12–1.90, = 0.005)
Smoking	5.12 (3.82–6.81, < 0.001)	2.91 (2.04–4.12, < 0.001)
Signs and symptoms		
Cough	1.19 (1.04–1.36, = 0.012)	–
Diarrhea	0.69 (0.56–0.83, < 0.001)	–
Fever	1.53 (1.34–1.76, < 0.001)	1.66 (1.43–1.94, < 0.001)
Headache	0.29 (0.26–0.34, < 0.001)	0.42 (0.36–0.49, < 0.001)
Runny nose	0.36 (0.31–0.42, < 0.001)	0.55 (0.46–0.65, < 0.001)
Shortness of breath	3.91 (3.45–4.44, < 0.001)	3.23 (2.81–3.71, < 0.001)
Sore throat	0.37 (0.31–0.44, < 0.001)	0.62 (0.51–0.75, < 0.001)

OR = odds ratio.

* *P*-value < 0.05 was considered statistically significant.

After adjustment for other variables, significant factors for odds of hospitalization were older age (3.40-fold greater odds); male gender (1.43-fold greater odds); Asian, indigenous, or unknown race (1.47-fold greater odds); any comorbidity (increased odds); fever (1.66-fold greater odds); headache (0.42-fold lower odds); runny nose (0.55-fold lower odds); shortness of breath (3.23-fold greater odds); and sore throat (0.62-fold lower odds) (Table 2). The multivariate analysis revealed that the comorbidity with higher odds for hospitalization was smoking (2.91-fold greater odds), followed by kidney disease (2.41-fold greater odds), obesity (1.74-fold greater odds), pulmonary disease (1.46-fold greater odds), diabetes (1.34-fold greater odds), and cardiovascular disease (1.30-fold greater odds) (Table 2).

### Death outcome.

Of the 10,713 patients, all 1,152 (10.8) who had been hospitalized were further analyzed for death by COVID-19. Chi-square analysis revealed that among the comorbidities analyzed in this study, only obesity (*P* = 0.334) and smoking (*P* = 0.053) showed no significant association with COVID-19 death (Table 3). All comorbidities were associated with higher death rates in hospitalized patients.

**Table 3:** Clinical characteristics of COVID-19 patients by outcome (hospitalized only)

	Survivor	Non-survivor	*P*-value*
Total, *N* (%)	696 (60.4)	456 (39.6)	
Age (years), *n* (%)			< 0.001
< 60	426 (78.0)	120 (22.0)	
≥ 60	270 (44.6)	336 (55.4)	
Gender, *n* (%)			0.275
Female	289 (58.5)	205 (41.5)	
Male	407 (61.9)	251 (38.1)	
Race, *n* (%)			0.190
White	217 (62.9)	128 (37.1)	
Black/multiracial	259 (57.2)	194 (42.8)	
Asian/indigenous/unknown	220 (62.1)	134 (37.9)	
Comorbidities, *n* (%)			
Cardiovascular diseases			< 0.001
No	426 (68.1)	200 (31.9)	
Yes	270 (51.3)	256 (48.7)	
Diabetes			< 0.001
No	561 (64.1)	314 (35.9)	
Yes	135 (48.7)	142 (51.3)	
Kidney diseases			0.001
No	673 (61.6)	420 (38.4)	
Yes	23 (39.0)	36 (61.0)	
Obesity			0.334
No	633 (60.9)	406 (39.1)	
Yes	63 (55.8)	50 (44.2)	
Pulmonary diseases			0.006
No	643 (61.8)	398 (38.2)	
Yes	53 (47.7)	58 (52.3)	
Smoking			0.053
No	658 (61.2)	417 (38.8)	
Yes	38 (49.4)	39 (50.6)	
Signs and symptoms, *n* (%)			
Cough			< 0.001
No	163 (48.8)	171 (51.2)	
Yes	533 (65.2)	285 (34.8)	
Diarrhea			0.001
No	602 (58.7)	424 (41.3)	
Yes	94 (74.6)	32 (25.4)	
Fever			< 0.001
No	166 (49.8)	167 (50.2)	
Yes	530 (64.7)	289 (35.3)	
Headache			< 0.001
No	472 (55.3)	382 (44.7)	
Yes	224 (75.2)	74 (24.8)	
Runny nose			< 0.001
No	523 (56.9)	396 (43.1)	
Yes	173 (74.2)	60 (25.8)	
Shortness of breath			< 0.001
No	370 (74.7)	125 (25.3)	
Yes	326 (49.6)	331 (50.4)	
Sore throat			< 0.001
No	567 (57.6)	417 (42.4)	
Yes	129 (76.8)	39 (23.2)	

* *P*-values were calculated by chi-square test. *P*-value < 0.05 was considered statistically significant.

All symptoms were also associated with the death outcome (*P* < 0.05) (Table 3), but only shortness of breath was more prevalent in non-survivors. Age was the only demographic characteristic associated with COVID-19 death (*P* < 0.001) (Table 3).

In univariate analysis, the odds of death were higher in patients aged 60 years or older, with any comorbidity (except obesity and smoking) and with shortness of breath, as well as lower if any other sign or symptom was presented (Table 4). After adjustment, the OR was significantly 3.95-fold greater for older patients and 3.55-fold greater for those with shortness of breath. Adjusted odds of death were significantly lower for patients who presented the following signs and symptoms: cough (0.59-fold lower), diarrhea (0.58-fold lower), headache (0.65-fold lower), and runny nose (0.66-fold lower) (Table 4).

**Table 4:** Risk factors associated with COVID-19 death in hospitalized patients

	Univariable OR (95% CI, *P*-value)	Multivariable OR (95% CI, *P*-value)*
Age (years) (vs. < 60)		
≥ 60	4.42 (3.42–5.73, < 0.001)	3.95 (2.95–5.33, < 0.001)
Comorbidities		
Cardiovascular diseases	2.02 (1.59–2.57, < 0.001)	1.26 (0.95–1.67, = 0.109)
Diabetes	1.88 (1.43–2.47, < 0.001)	–
Kidney diseases	2.51 (1.48–4.35, = 0.001)	1.68 (0.94–3.09, = 0.086)
Pulmonary diseases	1.77 (1.19–2.62, = 0.004)	–
Signs and symptoms		
Cough	0.51 (0.39–0.66, < 0.001)	0.59 (0.43–0.79, < 0.001)
Diarrhea	0.48 (0.31–0.73, = 0.001)	0.58 (0.36–0.93, = 0.025)
Fever	0.54 (0.42–0.70, < 0.001)	0.78 (0.58–1.05, = 0.100)
Headache	0.41 (0.30–0.55, < 0.001)	0.65 (0.46–0.91, = 0.012)
Runny nose	0.46 (0.33–0.63, < 0.001)	0.66 (0.45–0.95, = 0.029)
Shortness of breath	3.01 (2.34–3.88, < 0.001)	3.55 (2.68–4.73, < 0.001)
Sore throat	0.41 (0.28–0.60, < 0.001)	0.72 (0.47–1.11, = 0.140)

OR = odds ratio.

* *P*-value < 0.05 was considered statistically significant.

## DISCUSSION

In this study, we aimed to evaluate demographic and clinical characteristics to identify risk factors associated with COVID-19 hospitalization and death. For the analysis, data from the State Health Secretariats of Espírito Santo were retrieved, and independent risk factors for the outcomes were found using a multivariate binary logistic regression analysis.

Consistent with the previous literature,^7,17–23^ our study showed that elderly patients are generally more likely to be hospitalized and have a fatal outcome. The risk of hospitalization increased to 3.40-fold in patients aged 60 years or older (Table 2), and the risk of death was almost four times higher in older inpatients (Table 4). Although it is not yet clear why this relationship occurs, many explanations have been proposed by researchers, such as decrease in angiotensin-converting enzyme 2 (ACE2) protein expression in older individuals,^24^ more contact and developed immune response to other viruses,^25,26^ and higher prevalence of comorbidities.^27^ Angiotensin-converting enzyme 2 has been identified as the surface receptor responsible for SARS-CoV-2 entrance into cells.^28,29^ Deficiency of this protein is associated with a variety of conditions, such as older age, cardiovascular diseases, and diabetes. SARS-CoV-2 could affect this deficiency even more, accelerating, thus, inflammatory and thrombotic processes.^24^ Older patients might also have had more contact with other viruses, causing antibody-dependent enhancement and increasing inflammatory and immunologic responses.^25,26^ Last, there is a higher prevalence of chronic illness in older populations, even those not expressed in the database, such as neurological illness and inflammatory conditions.^27^ The older group had significantly more of the accounted conditions, except for obesity, contributing to overall worse health.

Several studies have shown that COVID-19–associated hospitalization rates have been higher among men than among women^22,23^ and that men die more from the SARS-CoV-2 infection than women.^19,30,31^ One possible explanation is that women’s immune response to the virus is stronger, and this could be attributed to protection by genes expressed on the X chromosome and sex hormones, which play an important role in innate and adaptive immunity.^32^ Another explanation is the presence of ACE2 on testes tissue and plasma at higher concentrations in men than women.^28,29^ This different expression in men could be a contributing factor for infection severity. However, these are only suppositions, as it is not clear if or why men are more at risk to die than women. In the present study, 13.7% of men versus 8.4% of women were hospitalized, and 38.1% hospitalized men versus 41.5% hospitalized women died. Gender was a risk factor only for hospital admission and not for death in hospitalized patients. Other reports also showed the equivalence of gender in COVID-19 death outcome.^18,33^

Racial and ethnic minority groups may be at a greater risk of serious complications from COVID-19 because of the increased prevalence of diabetes, cardiovascular disease, and other underlying health conditions.^34^ This was observed in the present study, with a higher prevalence of the following comorbidities in nonwhite patients: cardiovascular diseases (*P* < 0.001) and diabetes (*P* < 0.001). In multivariate analysis, being of Asian, indigenous, or unknown ethnicities was found to be an independent risk factor for hospitalization, increasing the odds by 1.47-fold. This adjusted increased risk may be caused by genetic differences in ethnicities^35^ (preprint). Once a patient was hospitalized, however, there was no difference by race in the odds of death.

In all, 39.6% of hospitalized patients were fatal cases, but this proportion was 7.7% for the entire dataset. This high proportion can be explained because of the selection of death and recovery cases because the hospitalized are already the ones in the worst condition. Higher death rates on hospitalized patients are also reported in other studies,^36,37^ and this not only speaks of the severity of these cases but unfortunately also shows that the intensive treatment is not very effective, mostly collaborating to increase life span to enable patient self-recovery. As noted by Dondorp et al.,^38^ COVID-19 pneumonia differs from pneumonia by other causative agents, and this could impact ventilation strategies and shows the need to look at alternative therapies because case fatality upon use of invasive ventilation is more than 50%. However, as the dataset does not disclose if patients used ventilators, no further conclusion can be drawn from this point.

Hospitalized non-survivors present a higher proportion of various coexisting chronic illnesses in univariate analysis. Previous studies reported similar findings.^17,36^ The most common comorbidities of COVID-19 patients in our cohort are cardiovascular diseases and diabetes, which is similar to that of previous studies.^17,30,39^ Among all 10,713 patients, of those who had cardiovascular problems, 17.5% died from COVID-19, whereas among those who did not have this comorbidity, the death rate was only 4.6%. Cardiovascular illness was confirmed to be an independent risk factor for hospital admission through multivariate binary logistic regression analysis, increasing odds of hospitalization by 1.30.

Kidney diseases, although present in only 1.2% of patients in the total dataset, was one of the comorbidities most strongly associated with hospital admission. Chronic kidney disease appears to be associated with an increased risk of serious COVID-19 infection,^40^ which may explain the high percentage of inpatients with kidney disease compared with those not hospitalized.

Smoking and obesity were two other comorbidities strongly associated with hospitalization. Although only 6.7% of those hospitalized are smokers, prevalence similar to that found by Farsalinos et al.,^41^ the percentage of smokers who were hospitalized was more than three times higher than that of hospitalized non-smokers. Smoking is already known as a risk factor for severe diseases such as respiratory infections.^42^ Obesity has previously been reported to be overrepresented in hospitalized patients with COVID-19^22^ and associated with hospitalization.^23,34^ Obesity is well recognized to be a pro-inflammatory condition,^43,44^ and induces diabetes and oxidant stress to adversely affect cardiovascular function.^45^ In the present study, 25% of obese people had diabetes (*P* < 0.001), and about 55% had cardiovascular problems (*P* < 0.001). Also, a lot of excess weight in the abdomen, below the diaphragm, makes breathing harder, which can restrict ventilation by decreasing diaphragm excursion. Obesity also impairs immune responses to viral infection.^46,47^ Although important in hospital admission, smoking and obesity did not increase the risk of death from COVID-19.

Cough and fever were the most frequent symptoms found in the present study as well as observed in previous studies.^17,18,31,33,39^ The overwhelming majority of all patients presented at least one of them (83.3%). In univariate analysis for death in hospitalized patients, all symptoms were important to explain the outcome. After adjustment, cough, diarrhea, headache, runny nose, and shortness of breath were significant to explain the outcome, and only shortness of breath increased the odds for death, with others being protective factors. Of the 10,713 patients, 18.2% of those who had shortness of breath died versus only 3.4% of those who did not have this symptom. Younger patients had more yet milder symptoms (cough, headache, runny nose, and sore throat). Older ones had a higher prevalence of shortness of breath, which was independently associated with worse prognostic.

It is also important to note that some signs, such as headache and sore throat, that rely on self-report, could be skewed on some age-groups who could not report them as efficiently, such as young children (younger than 4 years) or older adults with neurological problems or speech impairments (older than 80 years). This was not corrected in the data analysis, and reported signs and symptoms were evaluated as is in all age-groups.

Most recently published studies use only data from hospitalized patients. In the present study, when using notification data from the State Health Secretariat, we evaluated information from notified cases confirmed with COVID-19 and who presented an outcome of cure or death by SARS. We evaluated the outcome of death in hospitalized patients and the outcome of hospitalization for this disease.

This study has some limitations. First, it is important to highlight the underreporting of cases in Brazil, caused by the low availability of tests for extensive investigation of suspected cases, which may have generated less accuracy in the analyzes performed. Moreover, only patients with confirmed COVID-19 from Espírito Santo state were included; suspected but undiagnosed cases were ruled out from the analysis. It would be better to include as many patients as possible from Espírito Santo state, and from other Brazilian states, to get a more comprehensive understanding of COVID-19. However, many states do not make micro-data publicly available regarding disease cases collected by health secretariats.^13^

Second, in some cases, there was incomplete clinical information, which limited the use of some variables in the study and led to exclusion of incomplete observations, which may have caused bias in the estimation and reduced the representativeness of the samples. Besides that, more detailed patient information, particularly regarding laboratory parameters, was unavailable at the time of analysis. Espírito Santo Health Secretariat did not make openly available full clinical or laboratory data, and signs, symptoms, and comorbidities are not sufficient to evaluate a patient’s health condition, and many other laboratory parameters^48^ could affect hospitalization and mortality. However, the data in this study permitted an assessment of the epidemiological and clinical characteristics of 2019-nCoV infection in Espírito Santo state, Brazil.

Another limitation is that complex interactions involving many variables may not be correctly understood through multivariate binary logistic regression.^49^ For example, cardiovascular disease interacts in many ways with gender, symptoms, age, and other comorbidities, and these interactions may not be linear. This might lead to discarding important variables or assumptions about a certain variable being more important than what it actually is.

The study of odds of hospitalization and death could affect faster and differentiated care for those at higher risk of complications, and different prioritization of those with better prognostic, as it is increasingly common to find shortage of hospital beds and other resources.^11^

In conclusion, our study provided evidence that older age; male gender; Asian, indigenous, or unknown race; all comorbidities (smoking, kidney disease, obesity, pulmonary disease, diabetes, and cardiovascular disease, in this order), and fever and shortness of breath increased the risk of hospitalization. Headache, runny nose, and sore throat decreased this risk. For death outcome in hospitalized patients, older age and shortness of breath increased the risk, and cough, diarrhea, headache, and runny nose decreased the risk. Further study is needed to obtain a better understanding of the risk factors for death by COVID-19, incorporating other Brazilian states to identify possible differences in behavior in the different regions of a vast country such as Brazil.
